# Validity of air-displacement plethysmography in the assessment of body composition changes in a 16-month weight loss program

**DOI:** 10.1186/1743-7075-3-32

**Published:** 2006-08-22

**Authors:** Cláudia S Minderico, Analiza M Silva, Pedro J Teixeira, Luis B Sardinha, Holly R Hull, David A Fields

**Affiliations:** 1 Exercise and Health Laboratory, Faculty of Human Movement, Health and Exercise Science, Technical University of Lisbon, Portugal; 2 University of Oklahoma, Norman, OK, USA; 3 Department of Pediatrics, Children's Medical Research Institute's Metabolic Research Center, University of Oklahoma Health Science Center, OK, USA; 4 Assistant Professor, University of Oklahoma Health Science Center, School of Medicine Department of Pediatrics, OUCP Diabetes & Endocrinology, 940 NE 13^th ^Street, CH 2B2426, OKC, OK 73104, USA

## Abstract

**Objective:**

To compare the accuracy of air displacement plethysmography (ADP) and dual energy x-ray absorptionmetry (DXA) in tracking changes in body composition after a 16 month weight loss intervention in overweight and obese females.

**Methods:**

93 healthy female subjects (38.9 ± 5.7 yr, 159.8 ± 5.6 cm, 76.7 ± 9.9 kg, 30.0 ± 3.4 kg/m^2^) completed a 16 month weight loss intervention. Eligible subjects attended 15 treatment sessions occurring over the course of 4 months with educational content including topics relating to physical activity and exercise, diet and eating behavior, and behavior modification. In the remaining 12 months, subjects underwent a lifestyle program designed to increase physical activity and improve eating habits. Before and after the intervention, subjects had their percent body fat (%fat), fat mass (FM), and fat-free mass (FFM)) assessed by DXA and ADP.

**Results:**

Significant differences (p ≤ 0.001) were found between DXA and ADP at baseline %fat (46.0 % fat *vs*. 42.0 % fat), FM (35.3 kg *vs*. 32.5 kg) and FFM (40.8 kg *vs*. 44.2 kg) as well as at post intervention for %fat (42.1% fat *vs*. 38.3 % fat), FM (30.9 kg *vs*. 28.4 kg) and FFM (41.7 kg *vs*. 44.7 kg). At each time point, ADP %fat and total FM was significantly lower (p ≤ 0.001) than DXA while FFM was significantly higher (p ≤ 0.001). However, both techniques tracked %fat changes similarly considering that there were no differences between the two means. Furthermore, a Bland-Altman analysis was performed and no significant bias was observed, thus demonstrating the ability of ADP to measure body fat across a wide range of fatness.

**Conclusion:**

At baseline and post weight loss, a significant difference was found between ADP and DXA. However, the results indicate both methods are highly related and track changes in %fat similarly after a weight loss program in overweight and obese females. Additionally, the mean changes in %fat were similar between the two techniques, suggesting that ADP can be translated to its use in clinical practice and research studies as DXA currently is used.

## Background

It has been widely documented and reported the rise in obesity rates across the globe in all ethnicities and genders [1,2]. The link between obesity and its related co-morbidities and death is the concomitant increase in fat mass observed as body weight increases [3]. Accordingly, weight loss programs should not solely focus on decreasing body weight, but in addition to, focus on decreasing fat mass. Consequently, the need has arisen for accurate assessment tools in the management of obesity and in the evaluation and efficacy of weight loss programs. One such tool has been air-displacement plethysmography (ADP), in part because of its ability to accommodate large persons but also because of its ease on both the patient and operator [4-7].

Several studies have validated ADP with hydrostatic weighing, dual energy X-ray absorptiometry (DXA), bioelectric impedance, and multi-comportment models in a wide range of populations (children, elderly, athletes, morbid obesity, paraplegics) with the overall consensus showing good agreement [6,8-10]. Though warranted and needed, few studies have assessed the ability of ADP to track changes in body composition over time in persons engaged in a weight loss program [11,12]. Recently, Frisard *et al*. [11] concluded that ADP was relatively accurate in assessing body composition compared to DXA in a group of overweight males and females who engaged in a six month weight loss program, although ADP showed bias (i.e. it overestimated fat mass at body fat ranges <40%) This is in agreement with Weyers *et al*. [12]. who reported similar sensitivity between ADP and DXA in twenty-two subjects who were involved in a modest eight week weight loss program. Both the Frisard and Weyers studies analyzed a combined sample of men (22 and 10, respectively) and women (34 and 12, respectively) during a short period of weight loss (6 months and 8  weeks, respectively). Therefore, the purpose of this study was to determine the ability of ADP to track changes in percent fat (%fat), total fat (FM) and fat-free mass (FFM) in a 16 month weight loss program relative to DXA in a cohort of females.

## Methods

### Subjects

Subjects were recruited from the Lisbon community for a 16 month weight management program through newspaper advertisements, email messages, and study flyers. Inclusion criteria were the following: 1) female, 2) ≥ 24 years old, 3) pre-menopausal, 4) currently not pregnant nor trying to become pregnant, 5) body mass index (BMI) >24.9 kg/m^2^, and 6) free from any major diseases. After several orientation sessions, 152 females signed up for the weight loss program. During the run-in phase, four females decided not to participate (reporting time and scheduling conflicts), four did not comply with testing requirements, three females became pregnant or were attempting to become pregnant, and one subject was diagnosed with hyperthyroidism, leaving a total of 140 females who started the intervention. However, only 95 subjects completed ADP and DXA testing before and after weight-loss. An initial visit with the study physician ensured that subjects met all medical inclusion criteria. All participants agreed to refrain from participating in any other weight loss program and gave written informed consent prior to participation in the study. The Institutional Review Board of the Faculty of Human Movement approved the study.

### Weight loss intervention

As described elsewhere, subjects in the first phase attended 15 treatment sessions in groups of 32 to 35 women, for 4 months [13]. Average attendance to the treatment sessions was 83%. Sessions lasted 120 minutes and included educational content and practical application classroom exercises in the areas of physical activity and exercise, diet and eating behavior, and behavior modification. Physical activity topics included learning the energy cost associated with typical activities, increasing daily walking and lifestyle physical activity, planning and implementing a structured exercise plan, setting appropriate goals, using logs and pedometers for self-monitoring, and choosing the right type of exercises. Examples of covered nutrition topics are the caloric, fat, and fiber content, and the energy density of common foods, the role of breakfast and meal frequency for weight control, reducing portion size, strategies to reduce fat content in the diet, preventing binge and emotional eating, planning for special occasions, and reducing hunger by increasing meal satiety (e.g., increasing fiber content). Cognitive and behavioral skills such as self-monitoring, self-efficacy enhancement, dealing with lapses and relapses, enhancing body image, using contingency management strategies, and eliciting social support were also part of the curriculum. Subjects were instructed and encouraged to make small but enduring reductions in caloric intake and to increase energy expenditure to induce a daily energy deficit of approximately 300 kcal. Although weight was monitored weekly, subjects were advised that long-term (i.e., after 1–2 years), not necessarily rapid weight reduction was the primary target. In the first session, participants were informed that reaching a minimum of 5% weight loss at 6 months was an appropriate goal in this program and were subsequently instructed to individually calculate the number of kilograms that corresponded to their specific body weight. In the second phase subjects were involved in a lifestyle intervention through 12 month. Briefly, participants were asked to participate in a minimum of 4 and a maximum of 6 physical activity sessions weekly. Compliance was monitored on the basis of daily logs of physical activity. Monthly group sessions were used, with a minimum duration of 120 minutes. These group meetings were designed to support participants with positive experiences and to overcome individual and specific roadblocks to increase physical activity and improve nutrition.

### Body composition measurements

DXA was chosen as the criterion method which has been considered a reasonable alternative to a multi-compartment approach [11,14-16]. All subjects arrived for testing in the morning after a 12-hour fast. Additionally, subjects were asked to refrain from exercise, alcohol and stimulant consumption 24 h prior to testing.

#### Dual energy X-ray absorptiometry (DXA)

Each subject had %fat, FM, and FFM evaluated by DXA utilizing a whole body (QDR-1500, Hologic, Waltham, USA, pencil beam mode, software version 5.67 enhanced whole-body analyses) system. Prior to testing, the system was calibrated according to the manufactures recommendations. Following the protocol for DXA described by the manufacturer, a step phantom with six fields of acrylic and aluminum of varying thickness and known absorptive properties was scanned alongside each subject to serve as an external standard for the analysis of different tissue composition. The same lab technician positioned the subjects, performed the scans and executed the analysis according to the operator's manual using the standard analysis protocol. Based on ten subjects, the coefficient of variation (CV) and technical error of measurement (TEM) in our laboratory for %fat is 2% and 0.4% respectively.

#### Air-Displacement Plethysmography (ADP)

Each subject also had %fat, FM, and FFM evaluated by ADP (e.g. BOD POD, Life Measurement Incorporated, Concord, CA, USA, software version 1.68) according to manufacturer testing recommendations and guidelines. The system was calibrated every day according to the manufactures recommendations. Details regarding the physical concepts and operational principles of ADP are reported elsewhere [7,17]. Briefly, each subject wore a swimsuit and cap provided by the laboratory while body mass was measured to the nearest 100 g by an electronic scale connected to the ADP computer. Next, the measured thoracic gas volume was calculated where:

Thoracic gas volume = functional residual capacity + 0.5 tidal volume

The measured thoracic gas volume was obtained in all subjects. Body density (Bd) was then calculated as body mass divided by body volume. Percent body fat was estimated from body density based on a two-compartment model using Siri's equation [18]:

ADP %fat = [(4.95/Bd) - 4.50] × 100

Based on ten subjects, the CV and TEM in our laboratory for %fat is 3% and 1% respectively.

### Data analysis

Accuracy and bias were examined in ADP using DXA as the criterion method. Regression analysis was utilized to determine the accuracy of ADP. With ADP considered accurate if the regression between DXA and ADP did not have a slope significantly different from one and an intercept significantly different from zero. Additionally, R^2 ^and the standard error of the estimate (SEE) were assessed. Potential bias between ADP and DXA were examined using Bland-Altman analysis [19]. The Bland-Altman examined the difference between ADP and the criterion method (i.e. DXA) with a non-significant correlation indicating no bias in ADP across the degree of %fat loss. If subjects were > 3SD they were considered outliers and removed.

Data was analysed with SPSS for Windows version 14.0 (SPSS Inc, Chicago) and sstatistical significance was set at (p < 0.05).

## Results

The physical characteristics of all subjects who completed the study along with changes in body composition variables by both ADP and DXA before and after weight-loss are presented in (Table 1). Of note, two subjects were considered outliers (> 3SD), therefore the final sample for data analysis was 93 subjects.

**Table 1:** Subjects characteristic and body composition (n = 93).

**Variable**	**Before weight-loss**	**After weight-loss**	**Δ (post-before weight-loss)**
Age	38.9 ± 5.7		
Height (cm)	159.8 ± 5.6		
Weight (kg)	76.7 ± 9.9	73.17 ± 10.4	-3.6 ± 5.3**
BMI	30.0 ± 3.4	28.7 ± 4.0	-1.3 ± 2.1**
**DXA**			
%fat	46.0 ± 5.2	42.1 ± 6.4**	-3.9 ± 4.4
FM (kg)	35.3 ± 7.7	30.9 ± 8.4**	-4.3 ± 5.4
FFM (kg)	40.8 ± 4.4	41.7 ± 4.3**	0.95 ± 1.4
**ADP**			
%fat	42.0 ± 5.1^b^	38.3 ± 6.2^a^**	-3.7 ± 4.3
FM (kg)	32.5 ± 7.4^b^	28.4 ± 8.0^a^**	-4.1 ± 5.1^a^
FFM (kg)	44.2 ± 4.6^b^	44.7 ± 4.6^a^*	0.52 ± 1.6^a^

Data reported as mean ± standard deviation.

* Significantly different from baseline (p ≤ 0.05).

** Significantly different from baseline (p ≤ 0.001).

^a ^Significantly different from DXA (p ≤ 0.05).

^b ^Significantly different from DXA (p ≤ 0.001).

### Before and after weight-loss

ADP %fat was highly correlated with DXA %fat before weight-loss (r = 0.92) and after weight-loss (r = 0.94) (Table 2). There were significant differences in body composition variables between ADP and DXA before and after weight-loss (Table 1). At each time point (i.e. before weight-loss and after weight-loss), ADP %fat and total FM was significantly lower (p ≤ 0.001) than DXA while FFM was significantly higher (p ≤ 0.001).

**Table 2:** Summary of regression and Bland-Altman analysis before and after weight-loss compared to DXA

	Regression				Bland-Altman		
	Intercept	Slope	R^2^	SEE	Bias	95% Limits	*p *Value

**Before weight-loss**							
ADP %fat	6.667^1^	0.937	0.845	2.056	-4.012^3^	0.128 to 8.152	0.648
**After weight-loss (16 months)**							
ADP %fat	4.956^1^	0.969	0.884	2.183	-3.760^3^	0.600 to 8.120	0.408
**Δ (before – after weight-loss)**							
ADP %fat	-0.618^1^	0.902	0.763	2.182	0.252	-4.168 to 4.672	0.665

^1 ^Significantly different from 0 (p ≤ 0.05).

^2 ^Significantly different from 1 (p ≤ 0.001).

^3 ^Bland-Altman analysis bias is the mean difference between ADP and DXA (p ≤ 0.001) with the trend between DXA and ADP not significant at any time point (i.e. no bias between techniques was observed).

Accuracy of %fat was examined by the regression of %fat by DXA against %fat by ADP at each time point. A summary of the regression analyses between the relationship between %fat by DXA and ADP are presented in Table 2. The regression for %fat by ADP *vs*. %fat by DXA was significant only for the intercept (p < 0.05) for each time point (Table 2). Regression coefficients were above (R^2 ^> 0.85) and the SEE was low (< 2.2 %fat) for each time point as well (Table 2).

**Figure 1:**
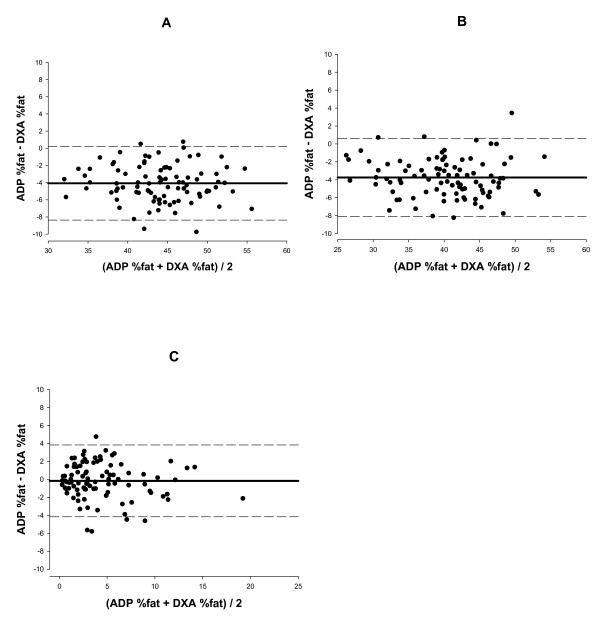
The Bland-Altman analysis at baseline (panel A), after weight-loss (panel B) and for the Δ (before – after weight-loss) (panel C). The middle solid line represents the mean difference between %fat from ADP – %fat from DXA and the upper and lower dashed line represents ± 2 SD from the mean i.e. 95% limits of agreement (± 1.96 SD). Bias between the techniques was not observed, as indicated by a non-significant p value (p = 0.648, p = 0.408 and p = 0.665, respectively).

Bland-Altman analysis was performed for each time point and in the Δ to determine if bias existed between ADP and DXA with plots shown in Figure 1 (panels A, B, and C respectively). A non-significant trend was observed for each time point, thus indicating no bias across the range of fatness.

### Δ (before and after weight-loss)

The Δ in %fat before and after weight-loss for ADP was highly correlated with DXA (r = 0.87) with a significant difference in the Δ in FM and FFM being observed (Table 1).

The regression in the Δ %fat by ADP *vs*. %fat by DXA significantly deviated from the line of identity (Table 2). The regression coefficient was (R^2 ^= 0.76) and the SEE was 2.2 %fat (Table 2).

**Figure 2:**
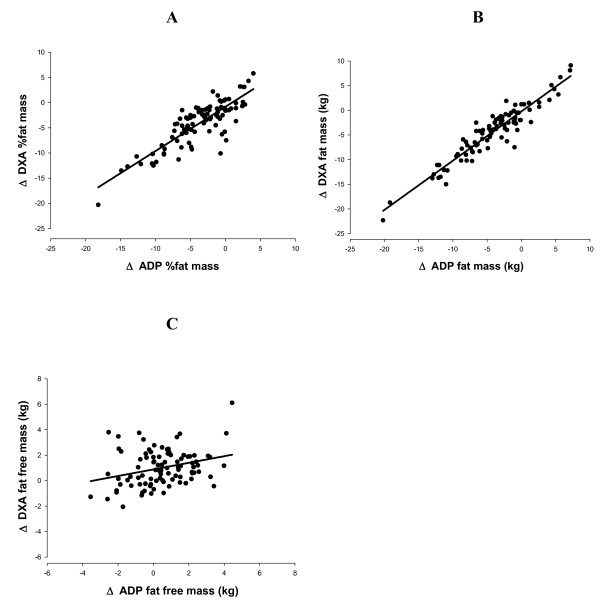
The relationships between the ΔADP and ΔDXA for %fat, fat mass (kg), and fat free mass (kg) are depicted in the scatter plots (A, B, and C, respectively).

The relationships between ΔADP and ΔDXA for %fat, fat mass (kg), and fat free mass (kg) are depicted in scatter plots shown in Figure 2 (panels A, B, and C respectively).

Bland-Altman analysis revealed a non-significant trend between the techniques, thus indicating no bias across the range of fatness (Table 2).

## Discussion

With the rapid rise in obesity worldwide, the focus has shifted to treatment of obesity which magnifies the necessity to assess changes in body mass accurately. Solely using body weight to evaluate weight loss outcomes could be misleading. It is imperative that methods to assess changes during weight loss have the ability to quantify changes in body weight such as changes in FM and FFM. ADP has emerged as a technique valid in several different populations and has the ability to accommodate larger subjects [4-7]. To date only two studies have examined the reliability of ADP over the course of a weight loss intervention (Weyers et al, Frisard et al.).

This study examined the accuracy and bias of %fat assessed by ADP relative to DXA before and after a sixteen month weight loss program. To our knowledge, this paper is unique due to its research design using a long-term weight loss program with a large sample of overweight and obese women.

### Discussion of techniques before and after weight-loss findings

The findings of our study indicate that there was a difference between ADP and DXA before and after weight loss for %fat, FM and FFM. ADP %fat and total FM was significantly lower than DXA while FFM by ADP was significantly higher than DXA FFM. Even though, pre and post weight loss measurements for %fat were significantly different, ADP %fat was highly correlated with DXA %fat and the SEE was low. Additionally, no bias was indicated by the Bland-Altman analysis demonstrating the ability of ADP relative to DXA to assess %fat across a wide range of fatness.

Two studies have investigated the ability of ADP to detect changes in body composition compared to DXA [11,12]. Weyers et al. tracked body composition changes in 12 overweight women and 10 overweight men after an 8 week moderate energy restricted diet [12]. In line with our study, Weyers et al. [12] found ADP to underestimate %fat and FM and overestimate FFM relative to DXA, at both time points. However, the study of Frisard et al. [11] reported the opposite. They randomized 56 overweight subjects into a self help group or a commercially available weight loss program [11]. Before and after weight loss, DXA results of %fat and FM were lower and FFM greater than ADP [11].

It is worth noting that our study used pencil-beam DXA technology (QDR-1500, Hologic, Waltham, USA), in which a single detector is used to measure the transmission of X-rays from a highly collimated source. Even though the difference between the pencil beam DXA and the multi-compartment model are relatively small, DXA slightly overestimates FM and underestimates FFM [20]. When compared to the new generation of fan-beam DXA with a slit collimator X-ray source and multiple detectors, and a different algorithm, the pencil-beam DXA gave a higher reading of FM and a lower value of FFM [21,22]. Differences in DXA instruments made by other manufacturers or differences in DXA instruments that use different scans modes and software is not known, though a few studies have shown a lack of inter-changeability in DXA systems to assess soft tissue [21,23-25].

Considering that Frisard et al used a fan-beam DXA (QDR 2000 Hologic), this may explain the lower cross sectional values for DXA %fat and FM before and after the intervention. Therefore, the different DXA technology utilized (pencil beam vs. fan beam) and the different algorithm used due to a new software version (1500 vs. 2000) might explain the discrepant results between the two studies. Although, Weyers et al, used different DXA equipment (ProdigyTM, Lunar Corporation, Madison, WI) and similar cross-sectional results were obtained compared to our study.

### Discussion of Δ (before and after weight-loss) between techniques

This study was specifically designed to determine if ADP tracked changes similarly to DXA. As assessed with a paired t-test, % fat changes were tracked similarly by both techniques because there were no differences between the two means, while FM changes were borderline significant (p = 0.049). Furthermore, a Bland-Altman analysis was completed and no significant bias was observed, thus demonstrating the ability of ADP to measure body fat across a wide range of fatness and that the techniques tracked body composition changes similarly.

Mentioned previously, two weight loss intervention studies have validated ADP with DXA in tracking body composition changes and have found similar results as this study [11,12]. After a 4.3 kg weight loss, data by Weyers et al. [12] found no significant differences in changes in %fat, FM or FFM between methods. Further, significant correlations between techniques were found for changes %fat and FM and no patterns in changes in %fat between ADP and DXA were detected. Frisard et al. [11] calculated regression coefficients comparing DXA and ADP after a 6.5 kg change in weight and found a high accuracy (r^2 ^>0.80) between the two techniques for %fat, FM and FFM.

In the current investigation, DXA was considered the reference method to validate ADP. However, DXA may not be accurate enough to detect changes in fat free mass components, due to the underlying assumption of the hydration of FFM for DXA. Moreover, our DXA-Hologic equipment performs whole-body scans using a pencil-beam mode which yields different results from other Hologic fan bean mode whole-body scans. In addition, the results of using this early software version compared to the new generation of Hologic DXA machines can be different. Therefore, the accuracy of ADP using this DXA equipment should not be generalized to other scan modes, software versions, and manufacturers (i.e. Lunar and Morland).

This study has several strengths including the large sample size, the length of intervention and the specific population studied. A total of 140 subjects started the intervention with 95 completing both the pre and post DXA and ADP measurements representing a 68% retention rate. A high retention rate is important because it strengthens the ability to identify the true relationship between ADP and DXA for detecting changes in body weight. Subjects were females greater than 24 years old, pre-menopausal with a BMI >24.9 kg/m^2^. The intervention lasted for a total of 16 months which is significant given that other studies have only assessed weight changes over a course of 8 week [12] or a 6 month intervention [11].

This study is not without limitations. First, the study population involved only females and may not be generalized to other populations such as children, males or the elderly. Second, the changes in body weight and body composition after the 16 month intervention were small (3.6 kg).

## Conclusion

The results of this study indicate that DXA and ADP are highly related and track changes in %fat similarly after a weight loss intervention in females. The mean changes in %fat were similar between the two techniques. However, before and after weight-loss, a significant difference was found between methods where ADP underestimated %fat and FM while overestimating FFM compared to DXA. DXA is a 3 compartment model where ADP is a 2 compartment model therefore methods of derivation of body fat are different which could contribute to the differences found between techniques. Both methods are relatively easy to complete with high subject compliance and both methods tracked changes in %fat similarly, therefore either could be used to track changes in body weight.

## Competing interests

The author(s) declare that they have no competing interests.

## Authors' contributions

CSM, AMS, PJT, and LBS were involved in the study design, data collection, and initial data analysis. All authors were involved in writing, editing and revising the manuscript and give their approval of this version for publication.
